# Heterogeneity in mammography use across the nation: separating evidence of disparities from the disproportionate effects of geography

**DOI:** 10.1186/1476-072X-7-32

**Published:** 2008-06-30

**Authors:** Lee R Mobley, Tzy-Mey (May) Kuo, David Driscoll, Laurel Clayton, Luc Anselin

**Affiliations:** 1 RTI International, 3040 Cornwallis Road, Research Triangle Park, NC, 27709-2194, USA; 2 Arizona State University, 600 East Orange Street, Tempe, AZ, 85287-0104, USA

## Abstract

**Background:**

Mammography is essential for early detection of breast cancer and both reduced morbidity and increased survival among breast cancer victims. Utilization is lower than national guidelines, and evidence of a recent decline in mammography use has sparked concern. We demonstrate that regression models estimated over pooled samples of heterogeneous states may provide misleading information regarding predictors of health care utilization and that comprehensive cancer control efforts should focus on understanding these differences and underlying causal factors. Our study population includes all women over age 64 with breast cancer in the Surveillance Epidemiology and End Results (SEER) cancer registries, linked to a nationally representative 5% reference sample of Medicare-eligible women located in 11 states that span all census regions and are heterogeneous in racial and ethnic mix. Combining women with and without cancer in the sample allows assessment of previous cancer diagnosis on propensity to use mammography. Our conceptual model recognizes the interplay between individual, social, cultural, and physical environments along the pathways to health care utilization, while delineating local and more distant levels of influence among contextual variables. In regression modeling, we assess individual-level effects, direct effects of contextual factors, and interaction effects between individual and contextual factors.

**Results:**

Pooling all women across states leads to quite different conclusions than state-specific models. Commuter intensity, community acculturation, and community elderly impoverishment have significant direct impacts on mammography use which vary across states. Minorities living in isolated enclaves with others of the same race/ethnicity may be either advantaged or disadvantaged, depending upon the place studied.

**Conclusion:**

Careful analysis of place-specific context is essential for understanding differences across communities stemming from different causal factors. Optimal policy interventions to change behavior (improve screening rates) will be as heterogeneous as local community characteristics, so no "one size fits all" policy can improve population health. Probability modeling with correction for clustering of individuals within multilevel contexts can reveal important differences from place to place and identify key factors to inform targeting of specific communities for further study.

## Background

Mammography is essential for early detection of breast cancer and both reduced morbidity and increased survival among breast cancer victims [1]. A lot of attention has been paid to low mammography use rates, and evidence of a decline in national rates over 2000–2005 has sparked renewed concern [2]. Evidence of persistent disparities in mammography use among women of different races and ethnicities is abundant [3]. A promising trend in public health research toward more multidisciplinary and multi-institutional research has predicated a new interest in examining community-level determinants of health disparities. Also, there is increasing recognition that health disparities may vary widely from place to place, such that the effects of place may be difficult to disentangle from social or cultural determinants of health [4-9].

Chandra and Skinner [6] argue that several factors are at work to confound the problem: there is considerable variation in health care utilization and outcomes across regions, minorities may use different providers than whites, and racial disparities may be higher in some areas. Together, these conditions create strong statistical interactions between geography and racial or ethnic identity, a fact that may lead researchers to falsely diagnose geographic variations as the determinant of racial disparities. Virnig et al. [5] show that disparities in several health care quality measures are wider *across *geographic regions than they are *within *the regions. Similarly, Coughlin et al. [7] contrast Southern counties with other counties in the United States and find that racial disparities in cancer screening are wider across counties than they are within them.

Relative homogeneity in socioeconomic conditions and health outcomes among people within regions contrasted with disparities in these things across regions suggests that spatial heterogeneity in such things as beliefs, practices, and resources may be a causal factor driving the observed disparities across regions. Probst et al. [4] argue that, because minorities tend to be concentrated more heavily in certain rural regions of the country, contextual factors that have impacted resource availability in those regions may produce worse health outcomes for all residents in those places. In this regard, Slifkin et al. [8] compare the health status of urban and rural minorities and find that several health status measures exhibit wide disparity between urban and rural minorities, specifically cancer screening and management, cardiovascular disease, and diabetes.

A better understanding of the socio-ecological factors impacting health outcomes is crucial, because a better balance of both the medical and nonmedical determinants of health is required to achieve "optimal" health outcomes. This theme is central to Smedley's recent commentary [10] regarding the necessity to focus on social and economic systems if we are to truly understand (and eradicate) health disparities. This literature supports the notion that place-specific resources, not minority status per se, may be driving some of the observed national health disparities statistics.

Clearly, disparities in health outcomes across races and ethnicities is a complex phenomenon with many determinants. Mervyn Susser [11] argues that traditional risk factor epidemiology has focused on a single level of analysis (the person or population) while ignoring social structures and dynamics that link individuals. Susser and Susser [12] advocate "eco-epidemiology" as a useful new paradigm for modern epidemiological research. This paradigm views the individual as existing within a set of nested constructs, where each level is part of a broader system and interacts with those above and below it. The eco-epidemiological paradigm has been embraced in public health research through multilevel modeling [13-25].

Multilevel modeling approaches have evolved over time from basic approaches using fixed effects to model higher-level data structures (in the absence of higher-order contextual data), to intermediate approaches that account for the redundancy in information from repeated higher-level contextual measures. Failure to account for the redundancy in information (i.e., the repeated county-level or PCSA-level variables for every woman in the county or PCSA) biases down the standard errors of the higher-order (county, PCSA) effect estimates. More complex approaches model the random effects of missing variables and covariances between individual-level effect parameters and higher-order (ecological) data structures [21]. There are two basic types of ecological effects: (1) a direct effect of a community-level variable on an individual-level health outcome; and (2) effect modification or interaction, whereby a community characteristic modifies the effect of an individual characteristic on an individual-level health outcome. In this paper, we investigate both types of effects using the intermediate approach to multilevel modeling, which includes many contextal measures at higher orders directly in the model and accounts for redundancies in these measures across individuals in areas by adjusting the standard errors using generalized estimating equations (GEE) clustering correction methods.

Public health researchers refer to area aggregates based on individual-level attributes reflecting characteristics of clients in an area as "collective" or "compositional" variables, to distinguish them from other ecological variables classified more broadly as "contextual" effects reflecting the nature of the physical or social environment [4,23,25,26]. Most public health research has included only collective effects in multilevel models, rather than contextual effects reflecting the broader political, cultural, social, or institutional expressions that affect access to and allocation of resources and opportunity [4,26,27]. For example, Litaker and Tomolo [28] and Litaker et al. [29] use an intermediate multilevel model like ours to model direct contextual effects and interactions between a woman's income and average income in her community on mammography use in Ohio. The studies by Litaker et al. are the only multilevel modeling studies we know of that attempt to estimate interaction effects for mammography use, and they find no significant effects. The limited geography or spatial homogeneity of ecological factors in Ohio may have impacted the significance of Litaker et al.'s findings.

The main objective of this paper is to use a multilevel modeling approach with a binary probability model of mammography use to examine various factors affecting mammography use in a large sample of women across 11 heterogeneous regions of the United States (Table 1). Our multilevel model is based on a carefully developed theoretical model (described below) of the ecological environment for mammography use. We posit that spatial heterogeneity in a variety of contextual factors, operating at different levels of influence, can help explain observed disparities within and across regions. We include a broad range of factors that include traditional access and health system supply variables, population/demand variables, and a rich set of socio-ecological variables describing other aspects of the community–besides health system and medical aspects–that are important correlates of observed behavior.

**Table 1:** Counts of women by sample category and age group in states

State	Total Number of Sample Women	Women in SEER Registry with Cancer Diagnosis	Women in 5% Medicare Sample, No Cancer Diagnosis	Women in Both SEER Registry and 5% Medicare Sample	Prop. Age 65–72	Prop. Age 73–80	Prop. Age 81 and Up
CA	70061	22773	47288	1141	0.34	0.33	0.33
CT	19194	8092	11102	417	0.30	0.38	0.31
GA*	8604	3117	5487	162	0.36	0.36	0.27
IA	17731	7343	10388	392	0.32	0.36	0.32
KY	15692	3092	12600	152	0.38	0.36	0.26
LA	13434	2705	10729	140	0.37	0.37	0.26
MI*	20980	8703	12277	425	0.33	0.39	0.28
NM	6524	2376	4148	124	0.32	0.39	0.29
NJ	30634	6600	24034	367	0.39	0.36	0.25
UT	7192	2820	4372	128	0.38	0.37	0.25
WA*	14539	6681	7858	357	0.34	0.37	0.29

* Only partial states are included in the analysis due to partial state coverage by the SEER registry.

### Conceptual model

Our conceptual model is a hybrid developed from several models from the behavioral health, socio-ecological, and health geography fields, as fully described elsewhere [9]. This spatial-interaction model conceptualizes the interplay between individual, social, and physical environments while delineating individual, local, and more distant levels of influence among compositional and contextual variables. The idea that the community factors influence human behavior is not new, but the explicit consideration of "what is the relevant zone of influence?" for ecological variables has only recently begun to appear in the literature [18,20,27,30,31].

**Figure 1:**
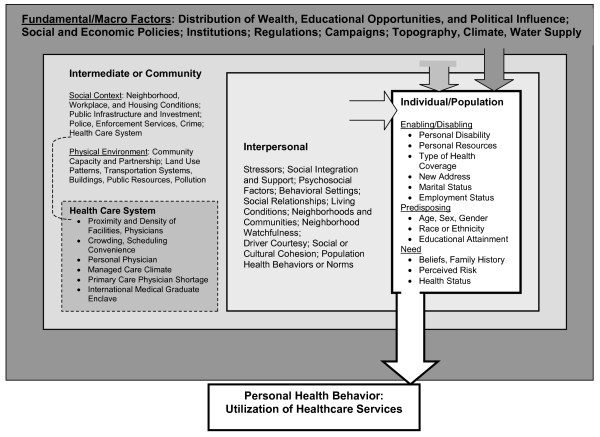
**Spatial interaction model of mammography utilization**. This spatial-interaction model conceptualizes the interplay between individual, social, and physical environments while delineating individual, local, and more distant levels of influence among compositional and contextual variables.

The conceptual model (Figure 1) positions the individual as making utilization choices (the final outcome) in a market context that has differentiated levels of influence for different classes of contextual variables and guides the selection of variables to be included in the analysis. The model also suggests the appropriate level of aggregation for compositional and contextual factors through its classification of the levels of influence. Some of the classification is derived from a synthesis of the body of literature cited herein; however, not all aspects we model have been considered in previous studies. The multilevel data provided by the authors as public use files should foster additional research in this fruitful area.

Individual characteristics are differentiated into the enabling, predisposing, and need constructs from the traditional Aday-Andersen behavioral health model [32]. The large box surrounding the entire figure represents the Fundamental, or macro-level, factors such as regulations, public policy, or media campaigns, represented at the state level in the hierarchy. These impinge upon the Intermediate community-level factors, such as characteristics of health care systems, land use patterns and development, crime, and housing conditions. Intermediate factors in turn impinge upon the Interpersonal neighborhood-level factors, such as social support, cultural cohesion, driver courtesy, and social capital. Interpersonal factors in turn impinge upon the Individual, who decides whether or not to use mammography.

We define the Intermediate, community-level factors at the county level, as these are the political units defined to manage the finances associated with community services. The Interpersonal, neighborhood-level factors should be defined at a smaller resolution than the community factors, but there are no guiding principles for defining these areal units from the literature. We developed data at various scales and thereby had the option to use either ZCTA or PCSA areal units to measure the Interpersonal factors. ZCTAs are U.S. Census ZIP code tabulation areas used to approximate the delivery area for a U.S. Postal Service five-digit ZIP code or collections of ZIP codes in urban areas, or three-digit ZIP codes in rural areas. Census 2000 long-form and population data are tabulated by the Census for these areal units. PCSAs are Primary Care Service Areas defined by Dartmouth College researchers for the Health Resources and Services Administration (HRSA), based on Medicare fee-for-service (FFS) patients' flows from home address to primary care physician offices, using ZIP code address of person [33]. In previous work, we found that using either ZCTAs or PCSAs as the areal units for the Interpersonal factors performed comparably [9]. We chose here to measure the Interpersonal factors at the PCSA unit, which has a natural preventive care market interpretation and is composed of one or more ZCTAs.

The variables used in this work and their sources are described in Table 2, which is divided into three sections corresponding to the conceptual model: Individual characteristics (categorized into enabling, predisposing, and need categories), Interpersonal (local neighborhood) factors, Intermediate (larger community) factors, and Fundamental (state level) factors. Table 3 gives sample statistics for women by state, and Table 4 gives sample statistics for the PCSA and county areas by state. We provide simple means, standard deviations, and the number of observations for each level of data (person, PCSA, county). If desired, the reader can convert the standard deviations to standard errors by dividing by the square root of sample size.

**Table 2:** Variables chosen for analysis, their contextual relevance, and sources

Characteristic of Sample Women	Data Source
*Enabling/Disabling*	Developed from linked SEER cancer registry and 5% Medicare data, provided by the National Cancer Institute [46].
Individual disability as original reason for Medicare entitlement	
Moved to a new residence in 2002–2003	
Months with extra assistance from state Medicaid, 2002–2003	
Distance to closest mammography facility	
*Predisposing*	
Age in 2002	Developed from linked SEER cancer registry and 5% Medicare data, provided by the National Cancer Institute [46]
Months enrolled in a Medicare HMO anytime in 2000–2001: prior period	
Race or ethnicity (white, African American, Asian, Hispanic, Native American, other)	
*Need*	
Had previous cancer diagnosis, breast or other cancers	
Obtained flu shot from doctor in 2002–2003	
Interpersonal Factors (PCSA)	Data Source
*Social Integration and Support*: Isolation index describing segregation by race or ethnicity (white, African American, Asian, Hispanic, Native American, other)	Developed from U.S. Census 2000 micro data at tract and ZCTA levels.
*Stressor, Driver Courtesy*: Commuter intensity reflecting the proportion of the workforce in each woman's residential area commuting 60 minutes or more each way to work	
*Social or Cultural Cohesion*: Proportion of the population in each woman's residential area immigrating into the United States between 1995–2000	Developed from U.S. Census 2000 micro data at tract and ZCTA levels.
*Social or Cultural Cohesion*: Proportion of the elderly in each woman's residential area with little or no English language ability	
*Stressor, Living Conditions*: Proportion of female elderly in each woman's residential area living alone	
*Stressor, Living Conditions*: Proportion of elderly in each woman's residential area living below the federal poverty level	
*Health Care System*: Number of mammography facilities per thousand elderly women in each woman's residential area, 2002–2003	Derived from SEER-Medicare linked database claims files [46] and U.S. Census.
*Health Care System*: Number of oncologists (or nurses) per thousand elderly, 2001 (2000)	Area Resource File
*Health Care System*: Ratio of counts of International Medical Graduates (IMGs) to U.S.-born physicians (generalists and specialists), 2000–2001	HRSA geospatial data warehouse
*Health Care System*: County designated as a primary care provider shortage area in whole or part of county, 2002	Data available from HRSA
*Health Care System*: Medicare managed care penetration, defined as proportion of the eligible population enrolled in Medicare HMOs, 2003	Centers for Medicare and Medicaid Services, Geographic Service Area Files
*Social Context*: Number of violent crimes known to police per thousand population	U.S. Department of Justice and FBI, National Archive of Criminal Justice Data
*Physical Environment*: Entropy index of land use mix: LANDMIX = - Σ_i = 1_^n ^Pi (ln Pi/ln n) Where n (5) is the number of different land use type classes in the county and P_i _is the proportion of land in type i in the county	Calculated by RTI using the 2001 National Land Cover Data (NLCD)

Notes: HMO = health maintenance organization; SEER = Surveillance Epidemiology and End Results; ZCTA = ZIP Code Tabulation Area; HRSA = Health Resources and Services Administration; ARF = Area Resource File; PCSA = Primary Care Service Area

**Table 3:** Sample statistics for person-level variables

	CA	CT	GA	IA	KY	LA	MI	NM	NJ	UT	WA	Range
Sample size	N = 70061	N = 19194	N = 8604	N = 17731	N = 15692	N = 13434	N = 20980	N = 6524	N = 30634	N = 7192	N = 14539	
Proportion with mammogram	0.59	0.66	0.61	0.63	0.54	0.54	0.64	0.52	0.58	0.61	0.64	0.52–0.66
Individual disability	0.062	0.047	0.062	0.044	0.084	0.066	0.061	0.061	0.056	0.043	0.050	.043–.084
	0.241	0.211	0.240	0.206	0.278	0.249	0.239	0.239	0.229	0.202	0.219	
Changed ZIP code	0.036	0.023	0.035	0.016	0.029	0.027	0.023	0.029	0.036	0.030	0.032	.016–.036
	0.187	0.149	0.183	0.126	0.167	0.162	0.148	0.168	0.185	0.170	0.176	
Months dual eligibility	5.773	2.534	3.116	2.056	4.143	5.106	2.097	3.829	2.648	1.282	2.260	1.28–5.77
	10.131	7.108	7.816	6.495	8.852	9.603	6.619	8.575	7.337	5.220	6.816	
Miles to closest facility	1.944	1.754	2.774	5.524	3.233	5.312	1.827	6.537	1.815	4.372	3.006	1.75–6.5
	3.944	2.552	3.484	7.058	4.804	7.664	2.191	13.607	2.802	8.743	4.911	
Age	76.538	77.107	76.116	77.095	75.909	75.947	76.544	75.704	76.752	75.840	76.505	75.8–77.1
	7.210	7.167	7.161	7.403	7.144	7.121	6.982	7.100	7.116	6.987	7.188	
Asian	0.069	0.005	0.004	0.001	0.001	0.002	0.005	0.002	0.007	0.006	0.021	.001–.069
	0.253	0.072	0.062	0.033	0.037	0.046	0.074	0.049	0.086	0.079	0.144	
African American	0.049	0.044	0.212	0.009	0.053	0.211	0.169	0.012	0.086	0.003	0.018	.003–.21
	0.215	0.206	0.409	0.095	0.225	0.408	0.375	0.107	0.280	0.058	0.133	
Hispanic	0.041	0.008	0.003	0.001	0.000	0.003	0.002	0.074	0.018	0.006	0.002	.000–.074
	0.198	0.088	0.053	0.033	0.014	0.055	0.047	0.262	0.132	0.078	0.046	
Native American	0.003	0.001	0.000	0.001	0.000	0.001	0.000	0.033	0.000	0.003	0.006	.000–.033
	0.057	0.025	0.015	0.030	0.011	0.030	0.015	0.179	0.020	0.051	0.075	
Other races or ethnicities	0.031	0.005	0.004	0.002	0.001	0.002	0.005	0.008	0.008	0.008	0.016	.001–.031
	0.173	0.068	0.064	0.041	0.038	0.050	0.073	0.086	0.087	0.088	0.127	
Diagnosed other cancer	0.027	0.049	0.033	0.054	0.015	0.014	0.047	0.033	0.017	0.039	0.047	.014–.054
	0.163	0.215	0.179	0.226	0.123	0.117	0.211	0.179	0.129	0.194	0.212	
Diagnosed breast cancer	0.325	0.422	0.362	0.414	0.197	0.201	0.415	0.364	0.215	0.392	0.460	.197–.46
	0.468	0.494	0.481	0.493	0.398	0.401	0.493	0.481	0.411	0.488	0.498	
Flu shot	0.544	0.589	0.617	0.517	0.577	0.613	0.598	0.503	0.597	0.667	0.599	.503–.667
	0.498	0.492	0.486	0.500	0.494	0.487	0.490	0.500	0.490	0.471	0.490	

Note: Sample means are provided on the first row for each variable, followed by sample standard deviations (SD) on the second row. Standard errors (SE) can be calculated using the sample size n from the first row, where SE = SD divided by square root of n.

**Table 4:** Sample statistics for area-level contextual variables

State	CA	CT	GA	IA	KY	LA	MI	NM	NJ	UT	WA
Number PCSAs	N = 335	N = 71	N = 22	N = 224	N = 146	N = 109	N = 18	N = 60	N = 140	N = 51	N = 63
Isolation index for African Americans	0.049	0.067	0.400	0.009	0.058	0.353	0.280	0.013	0.125	0.006	0.028
	0.078	0.104	0.195	0.026	0.094	0.171	0.327	0.015	0.158	0.014	0.046
Isolation index for Hispanics	0.310	0.077	0.061	0.023	0.015	0.020	0.049	0.501	0.121	0.095	0.050
	0.237	0.109	0.066	0.036	0.014	0.017	0.090	0.219	0.145	0.103	0.032
Isolation index for Asians	0.067	0.023	0.026	0.007	0.006	0.011	0.037	0.006	0.053	0.010	0.044
	0.085	0.015	0.029	0.009	0.010	0.031	0.032	0.008	0.055	0.011	0.053
Isolation index for Native Americans	0.022	0.004	0.003	0.004	0.004	0.012	0.004	0.154	0.002	0.042	0.051
	0.058	0.006	0.004	0.010	0.005	0.028	0.001	0.286	0.002	0.133	0.132
Isolation index for Pacific Islanders	0.003	0.001	0.001	0.001	0.000	0.001	0.001	0.002	0.001	0.004	0.004
	0.004	0.001	0.003	0.002	0.001	0.002	0.001	0.006	0.001	0.008	0.004
Proportion of workers commuting > 60 minutes to work	0.102	0.086	0.111	0.049	0.093	0.101	0.068	0.094	0.131	0.075	0.117
	0.059	0.060	0.030	0.026	0.048	0.041	0.032	0.069	0.055	0.047	0.080
Proportion of elderly with little or no English	0.286	0.152	0.246	0.074	0.084	0.083	0.192	0.249	0.243	0.182	0.184
	0.169	0.098	0.240	0.122	0.150	0.106	0.078	0.187	0.137	0.209	0.171
Proportion of population entering US 1995–2000	0.179	0.204	0.304	0.256	0.309	0.201	0.240	0.228	0.199	0.322	0.206
	0.070	0.078	0.171	0.191	0.212	0.153	0.086	0.161	0.087	0.141	0.096
Proportion of female elderly living alone	0.318	0.358	0.336	0.397	0.406	0.372	0.368	0.331	0.342	0.327	0.358
	0.067	0.053	0.053	0.049	0.049	0.058	0.044	0.081	0.067	0.076	0.062
Proportion of elderly in poverty	0.096	0.057	0.144	0.163	0.285	0.203	0.078	0.517	0.078	0.093	0.069
	0.238	0.042	0.093	0.872	0.949	0.122	0.054	2.693	0.086	0.095	0.032
Number counties	N = 58	N = 8	N = 15	N = 99	N = 120	N = 64	N = 3	N = 33	N = 21	N = 29	N = 13
Number of mammogram facilities per 1000 women	0.989	0.955	1.355	1.354	0.700	0.758	1.330	1.267	2.459	1.532	1.209
	0.313	0.297	1.354	1.686	0.767	0.368	1.119	1.364	3.936	1.884	0.700
Number of oncologists per 1000 elderly	0.099	0.093	0.033	0.088	0.039	0.167	0.028	0.019	0.046	0.082	0.087
	0.089	0.069	0.095	0.154	0.114	0.169	0.125	0.048	0.137	0.074	0.078
Number of nurses per 1000 elderly	7.077	4.145	1.896	2.736	5.150	2.976	1.884	3.359	1.898	4.843	3.394
	4.930	3.344	2.900	3.268	15.241	2.204	4.208	5.478	3.604	7.349	2.691
Ratio foreign born to U.S. doctors	0.248	0.354	0.255	0.222	0.232	0.304	0.136	0.204	0.063	0.187	0.088
	0.040	0.133	0.201	0.185	0.159	0.050	0.160	0.193	0.099	0.122	0.033
HMO penetration	0.205	0.294	0.096	0.041	0.094	0.066	0.197	0.174	0.094	0.124	0.103
	0.204	0.052	0.096	0.061	0.098	0.061	0.025	0.031	0.072	0.094	0.070
Violent crime rate	1.143	1.688	0.846	2.296	3.588	2.338	0.932	2.215	0.821	4.092	1.582
	0.687	1.110	0.711	1.999	1.733	2.447	1.068	1.541	0.514	1.302	0.668
Land use mix	0.212	0.470	0.367	0.192	0.192	0.190	0.893	0.560	0.042	0.083	0.282
	0.206	0.206	0.342	0.062	0.101	0.131	0.067	0.261	0.047	0.135	0.157
HRSA primary care physician shortage	0.828	0.875	0.867	0.475	0.675	0.641	0.667	0.381	0.879	1.000	0.923
	0.381	0.354	0.352	0.502	0.470	0.484	0.577	0.498	0.331	0.000	0.277

Note: Sample means are provided on the first row for each variable, followed by sample standard deviations (SD) on the second row. Standard errors (SE) can be calculated using the sample size n from the first row, where SE = SD divided by square root of n.

### Hypothesized associations between factors and mammography use

#### Individual characteristics

An important enabling characteristic is type of health coverage. All women in the sample are well-insured with traditional FFS Medicare Parts A and B insurance, which allow free choice of provider and mammography facility. Some women have additional coverage through a variety of state Medicaid programs for low-income or disabled elderly, which makes them dually eligible for Medicare and Medicaid insurance (which covers the Part B premium). We do not have information on individual income, but we assume that the dually eligible are lower-income than others in their state. Although there may be additional resources associated with dual eligibility, we hypothesize that disability and dual eligibility status are disabling characteristics, because physical limitations and poverty present additional burdens to care-seeking behavior. Shorter distance to the closest mammography facility is seen as an enabling characteristic. Another characteristic is recent address change–we hypothesize that moving is disruptive and a disabling characteristic. Predisposing factors included in the model are Medicare health maintenance organization (HMO) coverage in the 2 years prior to the study period. A recent mammogram might have been obtained under the HMO prior to joining FFS, which could lower the probability of utilization in 2002–2003. Also included are age and race or ethnicity. We include cancer diagnosis and utilization of flu shots as indicators of need. Individuals with a previous cancer diagnosis are more likely to experience another cancer, and mammography is used in the course of treatment as a diagnostic. Those utilizing flu shots are considered to have stronger health-seeking behavior.

#### Interpersonal factors

These include local neighborhood characteristics that impact one's perception of risk or information about health care through interactions with neighbors that shape opinions and beliefs. Communities may also provide support–both physical and psychological–for health-seeking behaviors. While residential segregation is often viewed as a harmful Fundamental factor–because it can influence the distribution of wealth, opportunity, and political influence toward the majority in the state–in the local neighborhood, residential segregation may impact social integration and support. We use Massey and Denton's [34] isolation index as our residential segregation measure, defined separately for each race or ethnicity relative to whites. These indices by race or ethnicity reflect the propensity for the minorities to come into contact with whites in residential neighborhoods. The index for a specific minority group ranges from 0 to 1, where higher values for the index reflects greater segregation among the minority from the white population. We hypothesize that the index may have positive impacts for some groups and negative impacts for others, because residential segregation effects have exhibited varied findings by race and ethnicity in the literature [35-38].

Two compositional variables are included to reflect social or cultural cohesion: the proportion of community members who have recently immigrated into the United States and the proportion of elderly community members with little or no English language ability. Both of these variables might reduce cohesion and the probability of mammography use. Several stressor variables are included for each woman's local community: commuter intensity, elderly women in poverty, and elderly women living alone. Inter-driver courtesy, which in our real-life experience decreases in communities with high commuter intensity, might affect the difficulty experienced by elderly who drive or for their caregivers who drive them. Areas with greater commuter intensity have been found to exhibit lower access to preventive care services among the elderly [39]; thus, we hypothesize that commuter intensity will reduce mammography use. We hypothesize that areas with higher proportions of elderly women living in poverty or living alone will exhibit lower mammography use rates due to lower social and material support and that women living in such areas will exhibit lower probability of utilization.

#### Intermediate community factors

At the wider community level are social context and physical environment factors that are affected by Fundamental resources that shape the infrastructure supporting community life [40]. Among these are characteristics of the health care system, such as physician shortage, facility density and proximity, and managed care climate. Managed care penetration in an area can change the way medicine is practiced, with spillover effects on FFS Medicare patients [41], so we hypothesize that women living in areas with greater managed care penetration may exhibit different probabilities of mammography use. Use would be higher if area attitudes among seniors regarding prevention were enhanced.

Availability of primary care physicians, medical oncologists, and nurses might increase the probability of mammography use. Women living in areas with primary care physician shortages might lower the probability of use. Primary care physician shortage is indicated using HRSA's measure at the county level. An alternative measure of physician availability is the ratio of International Medical Graduates (IMGs)–physicians of foreign origin who train in the United States–to native U.S.-born physicians. One study found that IMGs have disproportionately located in U.S. counties of greatest need, compared with native medical graduates, which reflects successful efforts through the J-1 visa waiver program [42].

We hypothesize that women living in counties with higher violent crime rates will be less likely to use mammography. There is considerable research examining the link between crime/disorder and fear [43] and evidence that fear may be limiting women's movement around their environments [44], especially for older women [45].

We include a land use mix index in the model to differentiate between sprawling suburbia, rural places, and mixed inner-city environments. The measure is an entropy index defined over the proportion of land in several different uses, at the 30-meter square level of resolution. The landmix measure is lower when there is more homogeneous use of land, so rural places and sprawling suburban housing developments have low values, and more urban areas with mixtures of homes and businesses have higher values. Because the more mixed environments are more urban in these data, we hypothesize that there will be a negative association between our landmix measure and probability of use if urban congestion impedes travel or reduces the desire to travel for care or if less congested rural settings were associated with improved probability of use.

## Methods

### Data and study sample

Our study population includes all women over age 64 with a breast cancer diagnosis in the Surveillance Epidemiology and End Results (SEER) cancer registries and a convenience sample of women over age 64 from the 5% Medicare file (see Table 1). The 5% Medicare file is linked by NCI to the SEER registry data and to all available Medicare claims [46]. All women over age 64 from the linked files who have Medicare claims and a valid address during the period 2002–2003 are included in our study population of 224,585 women.

The NCI linked SEER-Medicare database follows subjects longitudinally over the course of their remaining lives. NCI links the SEER registry data with a 5% sample of Medicare-eligible people residing in the SEER registry states. The 5% Medicare sample is nationally representative, drawn randomly from the 100% enrollment file containing all Medicare beneficiaries. People in the 5% file are drawn annually based on having specific digits in their Health Insurance Claim number (a permutation of the social security number), providing a nationally representative longitudinal sample that is useful as a reference sample for analysis of medical treatment paths, costs, utilization, and outcomes over time and comparisons between women with and without cancer [46]. This longitudinal feature allows us to use 2 years of claims data for each individual in our sample.

Some people in the 5% sample are also in the SEER registries because they have been diagnosed with cancer. In our work, we use women from the 5% sample (who may or may not have cancer) in combination with women from the SEER registries (who have been diagnosed with cancer). This combination of women with and without cancer diagnosis allows assessment of previous cancer diagnosis on propensity to use mammography. We include all women over age 64 with a breast cancer diagnosis rather than limiting the registry cohort to those also included in the 5% file. This sample design results in greater numbers of women with cancer diagnoses in the small areas that we study, allowing for a more robust inference.

NCI links to the registry and 5% Medicare subjects all available Medicare claims, providing information about the timing and type of mammography used. A recent study validates the use of Medicare claims data to assess mammography utilization [47]. We study women in the eight states (CA, IA, KY, UT, NM, LA, CT, NJ) and three portions of states (GA, WA, MI) covered by the SEER registries. Table 1 provides the counts of sample women in each state, by age, from SEER and 5% Medicare samples. The time period studied is the 2-year interval 2002–2003, and the outcome we study is any mammography use during this period.

Mammography utilization behavior is inferred from the Medicare claims files linked by NCI to the SEER registry and 5% Medicare reference subjects. However, only persons with traditional Medicare FFS coverage for both Part A (mandatory, covers hospitalizations) and Part B (elective, covers outpatient services) will have medical claims available for study. Other forms of health insurance, such as Medicare private insurance plans (Medicare HMOs, others) will produce no claims because these plans are not required to file claims with Medicare. While the vast majority of persons aged 65 and older have traditional Medicare FFS coverage, the proportion is dwindling and varies considerably across geography with the prevalence of Medicare private insurance plans [48]. Thus, the SEER-Medicare subsample we study is not nationally representative but conditional on a person's having traditional Medicare FFS coverage for both Parts A and B. Only a few women (less than 1%) were dropped from the analysis because their addresses could not be mapped to one of the SEER states due to bad or missing ZIP codes.

Lower-income or poor women are included in our study when they have coverage for Part B services, often achieved through dual eligibility for Medicare and Medicaid [48]. In our sample, the proportion of dually eligible varies from about 23% to 25% in CA and LA to less than 7% in UT, while about 17% on average over all areas. Thus, all women in our study sample had traditional, FFS Medicare, which allows choice of any provider and pays for annual mammograms.

### Geocoding methods

We obtained the ZIP code of address for each woman in the sample and first checked to see that the address from the enrollment database file was consistent with the address on the claims file. Enrollment file addresses are not updated continuously and women may migrate to other locations for services. For more accurate address location at time of service, when enrollment and claims addresses differed, we used the claims address as the valid address ZIP code.

We then calculated the longitude and latitude of the ZIP code boundary's centroid to determine which other areas (PCSA, county) to associate with each woman's address. In this geocoding process, we assumed that the ZIP code was associated with the census ZCTA or county that contained its centroid. This assumption was necessary because ZIP codes are not always neatly contained completely within ZCTA or county areas. Once all ZIP codes were associated with ZCTAs, finding the associated PCSAs was straightforward, because ZCTAs nest completely inside PCSAs. The contextual variables used for modeling were derived from census data defined at the ZCTA level or obtained from other PCSA- and county-level data sources (see Table 2).

### Multilevel data and empirical modeling

The multilevel data used in the empirical modeling were defined at the following levels: person, PCSA, and county. The fourth level, state, is omitted because there are not enough states to account for this level explicitly in the estimation. The multilevel data structure necessitates some form of multilevel modeling, and several alternatives are available. When there are not good measures of the contextual factors operating at different levels to include directly in the empirical model, researchers can model some of the unexplained place-specific variability using a random effects model specification [23]. However, when the place-specific heterogeneity is modeled directly through a rich set of covariates, the random effects variances often shrink to zero, and a random effects model specification is no longer necessary [49]. In this latter situation, the focus is on robustly estimating the effects of the higher-level covariates, which are repeated (redundant) over the lower units of analysis (i.e., women in the same county all are assigned the same HMO penetration variable). This redundancy can reduce the standard errors on the estimated coefficients of the higher-level variables, making them seem more statistically significant than they in fact are [49,21].

Because we have a very rich set of higher-order covariates, we use an intermediate modeling approach and focus on efficient estimation to produce reliable standard errors for the contextual covariates. We use the GEE robust empirical approach to correct the standard errors for biases stemming from redundancies in the contextual variables within areal units [50-52]. Horton and Lipsitz [51] note that in generalized linear models (GLMs) when the outcome variable is approximately normally distributed, standard likelihood approaches are useful for analysis of clustered data. To extend the GLM approach to models with discrete outcomes, such as our binary probit regression, Liang and Zeger [52] formulated the GEE approach, which is not likelihood-based and does not require parameterization assumptions for the second-order variance terms, which they refer to as a "working" matrix. The GEE approach is attractive because it provides a nonparametric, empirical approach that is robust to inappropriate assumptions about the variance-covariance matrix. The empirical approach is preferred when the number of clusters is large, which is a feature of our data (see Table 4) [51].

It is important to note that GEE estimators are used to characterize the average response for observations sharing the same set of covariates within an area (the unit of clustering) [51]. In our analyses, these GEE estimators provide efficient estimates of the association between a community contextual covariate and average women's response to it, in terms of the propensity to use mammography, in the community. Thus, we interpret the findings in the context of "women living in communities like X," rather than as specific community effects on individual women's behaviors.

We estimate binary probit regression models with factors representing the various levels in our contextual model: individual, neighborhood, and community. We examine direct effects of the neighborhood and community variables and interaction between individual-specific and contextual variables which we hypothesize will modify the direct effect estimates. The four interactions we examine are as follows:

1. individual's own race or ethnicity and same racial or ethnic segregation in her neighborhood (i.e., living in a segregated neighborhood among others of one's same ethnicity);

2. individual's age category (see Table 1) and managed care penetration in her community;

3. individual's age category (see Table 1) and commuter intensity in her neighborhood; and

4. individual's disability status and commuter intensity in her neighborhood, where disability status is determined by whether a women had personal disability as the original reason for Medicare entitlement.

Because of extreme multicollinearity between age or commuter intensity in interactions numbered 2 through 4 above, we are not able to include all interaction effects of interest simultaneously in one model. We estimate three separate models, all including the first set of interactions (own race and segregation) and one other set. Model 1 contains the disability by commuter intensity interaction, Model 2 includes the age group by managed care interaction, and Model 3 includes the age group by commuter intensity interaction. The three interaction models are presented side-by-side in the results (Table 5), by state. Age is kept as a continuous variable in Model 1 to estimate the linear effect of another year in age on probability of use. In Models 2 and 3, age is entered as a categorical variable to assess nonlinearities in the interaction term effects. The effect estimates that reach statistical significance at the 5% level or better are included in the body of Table 5. Effect estimates in Table 5 are interpreted as the effect of a one unit increase in the covariate on probability of mammography use in 2002–2003.

**Table 5:** Direct and interaction effects in mammography use: three specifications, by state

	Model 1	Model 2	Model 3	Model 1	Model 2	Model 3	Model 1	Model 2	Model 3
	POOLED	POOLED	POOLED	CA	CA	CA	CT	CT	CT
Individual disability	-0.07	-0.04	-0.04	-0.09	-0.03	-0.03	-0.06	-0.04	-0.04
Changed ZIP code	-0.05	-0.04	-0.04	-0.06	-0.06	-0.06	-0.05	-0.05	-0.05
Months dual eligibility	-0.01	-0.01	-0.01	-0.01	-0.01	-0.01	0.00	0.00	0.00
Miles to closest facility	0.00			0.00	0.00	0.00			
Age (Age group 2, age 73–80)	-0.02	-0.06	-0.05	-0.01	-0.08	-0.05	-0.02		-0.08
Age group 3, Age 81+	N/A	-0.25	-0.24	N/A	-0.25	-0.23	N/A	-0.23	-0.27
Asian	-0.05	-0.07	-0.07	-0.07	-0.06	-0.06			
African American	-0.03	-0.03	-0.03	-0.04	-0.04	-0.04			
Hispanic									
Native American	-0.06			-0.09	-0.09	-0.09			
Other races or ethnicities	-0.08	-0.06	-0.06	-0.07	-0.06	-0.06			
Diagnosed other cancer	0.06	0.06	0.06	0.07	0.06	0.06	0.04	0.04	0.04
Diagnosed breast cancer	0.31	0.33	0.33	0.30	0.30	0.30	0.29	0.30	0.30
Flu shot	0.17	0.18	0.18	0.17	0.17	0.17	0.18	0.18	0.18
Isolation index for African Americans	-0.01	-0.03	-0.03	-0.06	-0.07	-0.07			
Isolation index for Hispanics				-0.06	-0.05	-0.05			
Isolation index for Asians									
Isolation index for Native Americans									
Proportion of workers commuting > 60 min to work	-0.20	-0.06		-0.22	-0.18	-0.16	-0.19	-0.20	-0.23
Proportion of elderly with little or no English	-0.09	-0.06	-0.06	-0.06	-0.06	-0.06	-0.18	-0.18	-0.18
Proportion of elderly in poverty	-0.01			-0.11	-0.13	-0.12			
Number of mammogram facilities per 1000 elderly women		0.01	0.01	0.01	0.01	0.01			
Number of oncologists per 1000 elderly	0.07	0.08	0.08						
HMO penetration		0.09	0.08						
AfAmer*IsolationAfAmer	0.07	0.08	0.08						
Hispan*IsolationHispan							0.59	0.61	0.61
NativeAmer*IsolationNativeAmer		-0.17	-0.17	0.53	0.51	0.51			
Disability*LongCommute(1)		N/A	N/A	0.41	N/A	N/A		N/A	N/A
AGE2*HMO(2)	N/A		N/A	N/A	0.08	N/A	N/A		N/A
AGE3*HMO(2)	N/A		N/A	N/A		N/A	N/A		N/A
AGE2*LongCommute(3)	N/A	N/A	-0.20	N/A	N/A		N/A	N/A	
AGE3*LongCommute(3)	N/A	N/A	-0.14	N/A	N/A		N/A	N/A	

	Model 1	Model 2	Model 3	Model 1	Model 2	Model 3	Model 1	Model 2	Model 3
	GA	GA	GA	IA	IA	IA	KY	KY	KY
Individual disability	-0.30	-0.06	-0.06	-0.10			-0.08	-0.03	-0.03
Changed ZIP code				-0.10	-0.11	-0.11	-0.04	-0.05	-0.05
Months dual eligibility	-0.01	-0.01	-0.01	-0.01	-0.01	-0.01	-0.01	-0.01	-0.01
Miles to closest facility							0.00	0.00	0.00
Age (Age group 2, age 73–80)	-0.02			-0.02	-0.07	-0.05	-0.02	-0.10	-0.08
Age group 3, Age 81+	N/A	-0.28	-0.31	N/A	-0.28	-0.26	N/A	-0.31	-0.27
Asian									
African American									
Hispanic							N/A	N/A	N/A
Native American	N/A	N/A	N/A	-0.47	-0.44	-0.44	N/A	N/A	N/A
Other races or ethnicities									
Diagnosed other cancer	0.09	0.09	0.09	0.04	0.04	0.04	0.06	0.07	0.07
Diagnosed breast cancer	0.30	0.31	0.31	0.30	0.30	0.30	0.38	0.38	0.38
Flu shot	0.18	0.18	0.18	0.12	0.12	0.12	0.18	0.18	0.18
Isolation index for African Americans				-0.23	-0.23	-0.23	0.07		
Isolation index for Hispanics				-0.27	-0.24	-0.23			
Isolation index for Asians									
Isolation index for Native Americans									
Proportion of workers commuting > 60 min to work				-0.54	-0.48				
Proportion of elderly with little or no English				-0.07	-0.07	-0.07			
Proportion of elderly in poverty									
Number of mammogram facilities per 1000 elderly women				-0.01	-0.01	-0.01			
Number of oncologists per 1000 elderly				0.11	0.11	0.11	0.13	0.13	0.13
HMO penetration								-0.10	
AfAmer*IsolationAfAmer									
Hispan*IsolationHispan							N/A	N/A	N/A
NativeAmer*IsolationNativeAmer	N/A	N/A	N/A	4.33	4.07	4.07	N/A	N/A	N/A
Disability*LongCommute(1)	1.89	N/A	N/A		N/A	N/A		N/A	N/A
AGE2*HMO(2)	N/A		N/A	N/A		N/A	N/A		N/A
AGE3*HMO(2)	N/A		N/A	N/A		N/A	N/A	0.14	N/A
AGE2*LongCommute(3)	N/A	N/A		N/A	N/A		N/A	N/A	
AGE3*LongCommute(3)	N/A	N/A		N/A	N/A		N/A	N/A	

	Model 1	Model 2	Model 3	Model 1	Model 2	Model 3	Model 1	Model 2	Model 3
	LA	LA	LA	MI	MI	MI	NJ	NJ	NJ
Individual disability		-0.04	-0.04	-0.19	-0.06	-0.06	-0.07	-0.07	-0.07
Changed ZIP code				-0.10	-0.10	-0.10	-0.03	-0.04	-0.04
Months dual eligibility	0.00	0.00	0.00	-0.01	-0.01	-0.01	0.00	0.00	0.00
Miles to closest facility	0.00	0.00	0.00						
Age (Age group 2, age 73–80)	-0.02	-0.06	-0.13	-0.02	-0.15		-0.02	-0.11	-0.09
Age group 3, Age 81+	N/A	-0.25	-0.27	N/A	-0.42	-0.21	N/A	-0.33	-0.26
Asian							-0.07	-0.06	-0.06
African American							-0.04	-0.03	-0.03
Hispanic							0.06		
Native American				N/A	N/A	N/A			
Other races or ethnicities	-0.19	-0.19	-0.18				-0.08	-0.07	-0.07
Diagnosed other cancer	0.10	0.11	0.11	0.08	0.08	0.08	0.06	0.06	0.06
Diagnosed breast cancer	0.38	0.38	0.38	0.29	0.29	0.29	0.41	0.41	0.41
Flu shot	0.18	0.18	0.18	0.14	0.14	0.14	0.20	0.20	0.20
Isolation index for African Americans									
Isolation index for Hispanics							0.07	0.08	0.08
Isolation index for Asians									
Isolation index for Native Americans									
Proportion of workers commuting > 60 min to work			-0.54	-0.53			0.14	0.12	
Proportion of elderly with little or no English							-0.18	-0.18	-0.18
Proportion of elderly in poverty									
Number of mammogram facilities per 1000 elderly women				0.17	0.35	0.19			
Number of oncologists per 1000 elderly			0.07		-0.70				
HMO penetration				N/A	N/A	N/A			
AfAmer*IsolationAfAmer							0.11	0.12	0.12
Hispan*IsolationHispan							0.17	0.19	0.19
NativeAmer*IsolationNativeAmer				N/A	N/A	N/A			
Disability*LongCommute(1)	-1.03	N/A	N/A	1.87	N/A	N/A		N/A	N/A
AGE2*HMO(2)	N/A		N/A	N/A	N/A	N/A	N/A		N/A
AGE3*HMO(2)	N/A		N/A	N/A	N/A	N/A	N/A		N/A
AGE2*LongCommute(3)	N/A	N/A	0.78	N/A	N/A		N/A	N/A	
AGE3*LongCommute(3)	N/A	N/A		N/A	N/A	-0.90	N/A	N/A	

	Model 1	Model 2	Model 3	Model 1	Model 2	Model 3	Model 1	Model 2	Model 3
	NM	NM	NM	UT	UT	UT	WA	WA	WA
Individual disability	-0.15	-0.05	-0.05						
Changed ZIP code									
Months dual eligibility	-0.01	-0.01	-0.01	-0.01	-0.01	-0.01	-0.01	-0.01	-0.01
Miles to closest facility				0.00	0.00	0.00			
Age (Age group 2, age 73–80)	-0.02	-0.08	-0.06	-0.02			-0.01	-0.08	-0.07
Age group 3, Age 81+	N/A	-0.29	-0.23	N/A	-0.26	-0.24	N/A	-0.21	-0.24
Asian							-0.07	-0.07	-0.07
African American									
Hispanic	-0.17	-0.17	-0.17						
Native American									
Other races or ethnicities				-0.12			-0.08	-0.07	-0.07
Diagnosed other cancer							0.03		
Diagnosed breast cancer	0.32	0.32	0.32	0.29	0.29	0.29	0.24	0.24	0.24
Flu shot	0.13	0.13	0.13	0.15	0.15	0.15	0.22	0.22	0.22
Isolation index for African Americans							-0.19	-0.20	-0.20
Isolation index for Hispanics	-0.22	-0.20	-0.20						
Isolation index for Asians	-2.81	-2.59	-2.56						
Isolation index for Native Americans	-0.09	-0.08	-0.08						
Proportion of workers commuting > 60 min to work									
Proportion of elderly with little or no English				-0.16	-0.14	-0.14			
Proportion of elderly in poverty									
Number of mammogram facilities per 1000 elderly women									
Number of oncologists per 1000 elderly							0.22	0.21	0.21
HMO penetration	0.40		0.40						
AfAmer*IsolationAfAmer							0.88		
Hispan*IsolationHispan	0.26								
NativeAmer*IsolationNativeAmer	-0.31	-0.31	-0.31						
Disability*LongCommute(1)	1.41	N/A	N/A		N/A	N/A		N/A	N/A
AGE2*HMO(2)	N/A		N/A	N/A		N/A	N/A		N/A
AGE3*HMO(2)	N/A	0.45	N/A	N/A		N/A	N/A		N/A
AGE2*LongCommute(3)	N/A	N/A		N/A	N/A		N/A	N/A	
AGE3*LongCommute(3)	N/A	N/A		N/A	N/A		N/A	N/A	

Notes: N/A = not applicable for the model reported (e.g., cells associated with interactions not included in a particular model). Blank (white) cells in the table appear for estimates that were not statistically significant at the 5% level or better.

We propose several hypotheses. If living in a segregated community among one's own race or ethnicity increases social support, the interaction effect on mammography use will be positive. If disability is associated with enhanced transportation alternatives, then disabled women may be less affected by commuter conditions than other women. If FFS-insured Medicare beneficiaries closer to retirement age (age 65–72) are less affected (i.e., more independent in dictating their own health care activities) by market conditions (managed care practice spillovers) than older beneficiaries, then the effect of managed care spillovers will be greater for older beneficiaries (age 73–80, 81+) than younger beneficiaries (age 65–72). If advancing age makes one less affected by commuter intensity, the effect of commuter intensity will be less significant for older than younger elderly groups. This may happen if women of younger age (65–72) are still driving themselves, compared with the older age groups (73–80, 81+).

To assess these hypothesized relationships, we estimate each state as a separate region, a strategy that allows the maximum amount of heterogeneity possible, as the regression slopes can vary from state to state. We contrast the findings from the state-specific models with a pooled model containing state-specific dummy variables, which forces each effect estimate to be the same across states. The comparison is used to suggest how misleading a pooled approach (which ignores spatial heterogeneity) can be when examining disparities in mammography use. However, because the binary probit GEE models are estimated using maximum likelihood methods, there is no way to conduct a statistical test of the observed differences in the estimated effect parameters across the state-specific models (for those models with identical specifications, which include all states except MI, GA, KY). The pooled models cannot be used to assess meaningful differences across states either, because the state-specific dummies only capture differences in the average probability of mammography use across the states, forcing all of the effect estimates to be the same across states. Thus, we can provide descriptive comparisons only for differences noted across states.

The sample size in states varies. Also, MI, GA, and WA samples only contain the substate portions of those states covered by the SEER registries (see Table 1). The GA sample spans urban and rural areas in GA and is larger than the UT or NM samples, which cover those entire states. The WA sample also covers urban and rural areas, but the MI sample is very urban, covering the tri-county Detroit metropolitan area. All other states have both urban and rural areas and complete statewide coverage. Results from Michigan are not strictly comparable to other states because the three Detroit metropolitan area counties exhibit too little variation for all of the county-level variables to be included in the model. Managed care penetration and provider supply variables were correlated more than 95% using simple Pearsonian correlations; only two county-level variables–number of mammography facilities and oncologists per capita–were sufficiently uncorrelated to allow inclusion. Also, so few Native American women were present in GA, KY, and MI that they were pooled into the "other race" category and the interaction between Native American race and area segregation could not be included for these states. Similarly, the Hispanic ethnicity and its interaction with segregation were not possible in KY. Thus, the model specifying interaction between race or ethnicity and isolation of that same race or ethnicity is not uniform across states due to data limitations.

Several variables included in the estimation and in the sample statistics presented in Tables 3 and 4 were dropped from the reported results (see Table 5). Their estimated impacts were not significant in one or more states, so for brevity we dropped proportion of elderly women living alone, violent crime rate, land use mix, the ratio of international medical graduates to U.S.-trained physicians (these were only significant in CA, see [9]); and proportion of the population moving into the area 1995–2000 (significant in UT, effect estimate 0.21 with p-value 0.01), and the binary indicator of primary care physician shortage (not statistically significant anywhere). We also dropped a significant variable, number of nurses per thousand elderly, which had a significant but tiny positive effect in the pooled, CA, and NJ models (the effect was 0.00 for a 1-nurse increase per 1,000 elderly). One statistically significant individual-level control variable was also dropped for brevity: the number of months enrolled in a Medicare HMO in the 2 years prior to the study. The estimated effect was tiny, negative (-0.00), and statistically significant in CA, CT, NJ, and WA reflecting the fact that women in an HMO plan prior to the period under study may have received recent mammography that very slightly lowered their probability of use in the period studied. Full results are available from the authors upon request.

## Results

Table 5 contains the multilevel regression results from the pooled and state-specific models. Only the effects that are statistically significant at the 5% level or better are presented in the table. The pooled model contains state-specific intercepts that suggest lower average probabilities of mammography use in all states relative to CA, the reference state, after adjusting for other covariates. These state-specific intercepts from the pooled models (in the first three columns of Table 5) are presented in Table 6.

**Table 6:** Pooled model state-specific effects (model intercept estimates)

Washington	-0.05	-0.02	-0.02
Georgia	-0.05	-0.02	-0.03
Utah	-0.10	-0.08	-0.08
Connecticut	-0.01	-0.01	-0.01
New Jersey	-0.07	-0.04	-0.05
Kentucky	-0.08	-0.04	-0.04
Louisiana	-0.07	-0.02	-0.03
Iowa	-0.04	-0.04	
Michigan	-0.04		
New Mexico	-0.06	-0.03	-0.04

Notes: California is the omitted reference group. Blank (white) cells in the table appear for estimates that were not statistically significant at the 5% level or better.

Individual-level effects are fairly consistent across interaction models and states. Disability is associated with significantly lower probabilities of mammography use, ranging from about 3% to 7% lower probability across states. Dual eligibility effects are smaller, amounting to at most 1% lower probability in all states. Use of mammography declines about 1% to 2% with each additional year of age (Model 1) and declines more with higher age categories (Models 2 and 3). Having recently moved to a new ZIP code is associated with lower probability of use in six states, reaching a substantial 10% to 11% lower probability for IA and MI. Distance to closest provider effects are quite small and only present in four states: CA, KY, LA, and UT. Flu-shot behavior is associated with about 12% to 22% higher probability of mammography and is significant across all states, suggesting that women who receive flu shots from their doctors are also more likely to utilize mammography. Having a cancer diagnosis is associated with a much higher probability of use in every state. With so much agreement across states, the pooled model results are quite consistent with the state-level findings in terms of size and sign of effect for these individual-level variables. Individual's race or ethnicity effects, where statistically significant, are negative, suggesting that women of other races and ethnicities are less likely to use mammography than white women.

Turning to the neighborhood variables, the effect estimates associated with the segregation indices vary in numerical sign across states. On average, women living in more segregated communities appear to have lower probability of use in some states and higher probability in others. The pooled effect estimates reflect this variability across states, often not achieving statistical significance. Women living in more segregated African American communities appear to have lower probabilities of mammography use in CA, IA, and WA but may have higher probability in KY (Model 1 only). Women living in more segregated Hispanic communities appear to have lower probabilities of use in CA, IA, and NM, but higher probabilities in NJ. Women living in more segregated Asian or American Indian/Alaska Native communities appear to have lower probability of use in NM only.

Residential segregation in the immediate neighborhood is a measure of cohesion; however, to understand social support, we examine whether women of a particular race or ethnicity living in same-race segregated communities are more or less likely to use mammography. If living among others of the same race or ethnicity encourages healthier behaviors, then there would be a positive effect from this interaction on mammography use. For African American women, the effect is positive in NJ, MI, and WA (Model 1 only) and also positive in the pooled model. For Hispanic women, the effect is positive in CT, NJ, and NM (Model 1 only). For Native Americans, the effect is positive in CA and IA but negative in NM and in the pooled estimate. We note that when the sign of the effect varies across states, the pooled model will inevitably contradict some state findings and agree with others, when it achieves significance.

Several PCSA-level variables had significant effects. Commuter intensity–the proportion of the workforce in a local neighborhood who commuted more than 60 minutes each way to work–is a large negative effect on the probability of use in CA, CT, IA, LA, and MI. The effect is positive in NJ, suggesting that elderly women living in commuter communities there are actually more likely to use mammography. Interaction between age and commuter-intensity (Model 3) suggests that older women living in commuter intense areas are less likely to use mammography than the younger elderly group (age 65–73), in the pooled model and in MI. By contrast, in LA, older women living in more commuter-intense areas are more likely to use mammography than younger elderly women. In the interaction between commuter intensity and disability status (Model 1), evidence suggests that disabled women living in commuter-rich communities are more likely to use mammography in CA, GA, MI, and NM but less likely in LA; the pooled results reflect the findings for LA only.

Living in communities where proportionately more elderly have little or no English language ability is associated with lower utilization in five states (CA, CT, IA, NJ, and UT), consistent with the pooled results. Living in communities where proportionately more elderly are impoverished is associated with lower utilization in CA only, where the effect is larger than the language ability variable.

Health services provider variables such as mammography facilities and oncologists per thousand older persons have significant positive effects in the pooled sample and in some states but negative effects in others. Oncologist supply is associated with significantly higher use in the pooled model, IA, KY, LA (Model 3 only), and WA. However, having a greater number of facilities available per capita is associated with lower utilization in IA but higher utilization in MI and CA; effects are quite large in MI.

Area HMO penetration is a significant negative predictor in KY (Model 2 only) but is positive in NM and in the pooled average. Positive HMO effects are consistent with change in area behavior toward greater utilization of preventive care services where there is greater HMO penetration. Interaction effects with HMO penetration and age suggests that older women are more likely (than those in the age 65–72 cohort) to use mammography in HMO-rich markets in CA, KY, and NM.

## Discussion

### Limitations

It is well known that the race and ethnicity coding in the Centers for Medicare & Medicaid Services (CMS) Enrollment Data Base (EDB) is not perfect, with a greater degree of error for persons who are not white or African American [53,54]. However, large administrative databases are thought to be more reliable sources of population race or ethnicity data than small household or other surveys, which typically underrepresent minorities [54]. In efforts to improve and expand the coding, CMS conducted a postcard survey of over 2 million beneficiaries with Hispanic surnames or countries of origin in 1997 and beneficiaries with "other" or "missing" race or ethnicity codes. The survey resulted in updated coding for about 858,500 beneficiaries, improving the race or ethnicity coding of the EDB, as the number of persons with a race code of "other" or "unknown" decreased from 978,000 in 1993 to 473,000 in 1997 [53,54]. An analysis comparing the distribution of race or ethnicity for Medicare beneficiaries aged 65 or over as coded in the updated EDB to that of U.S. Census estimates for the same age group found very similar distributions, concluding that studies of disparities in utilization rates using the EDB as the source of racial or ethnic coding would likely provide unbiased estimates of these utilization rates [54]. However, because the race and ethnicity coding is less reliable for counts of individuals who are not white or African American, our findings for these groups should be interpreted with caution and validated in future research.

Differences in findings across states may derive in part from the different sample sizes and the spatial sufficiency of samples within states, and we cannot test for this in these data. Also, in the single time interval examined here, this cross-sectional analysis is limited to suggesting evidence of associations, not causal relationships. Another limitation is the ability to generalize the policy findings to other areas or states. Hence, our results should be interpreted as descriptive findings, which may offer some insights into areas of potentially fruitful further study.

### Summary of findings

In this paper, we examine various factors affecting mammography use and disparities in use from a large random sample of women aged 64 or older with traditional Medicare coverage across 11 heterogeneous regions of the United States. We estimate a binary probability model of mammography use, with multilevel modeling to account for redundancies in higher-level contextual variables assigned to women within geographic units. Our multilevel model is based on a carefully developed theoretical model of the ecological environment for mammography use. The theory posits that a variety of contextual factors, operating at different levels of influence, can help explain observed mammography use. Factors include traditional access and health system supply variables, population/demand variables, and a rich set of socio-ecological variables describing other aspects of the community–besides health system and medical aspects–that are important correlates of observed behavior.

We find considerable variation among states in the effect estimates of contextual variables with some policy implications. Commuter intensity seems to deter mammography use in some states and increase it in another. Given this variability in findings across the states, a transportation policy aimed at improving access to mammography for elderly women in commuter communities should perhaps be tailored to serve regions where commuter crowding is apparently an impediment. Elderly living in communities with worse English language ability among the elderly are less likely to use mammography in five states. This finding suggests that health communication policy to increase health literacy may be needed in some communities where the elderly are isolated due to poor language abilities. Women living in impoverished elderly communities are less likely to use mammography, although all have both Parts A and B of traditional Medicare coverage, allowing free choice of provider and annual mammograms free of charge. This finding suggests that impoverished environs are important determinants of mammography utilization even for those residents with the means to pay for mammography services. Prevalence of HMOs is positively associated with utilization in some states and negative in others. The impact of HMO spillovers on preventive care behaviors is not consistent across states and seems greater for older women. Because the tendency is to utilize mammography less with increasing age, the HMO spillovers seem to counter this trend in a few states.

## Conclusion

The main objective of this paper is to explore the hypothesis that regression models estimated over pooled samples of heterogeneous states may provide misleading information regarding predictors of health care utilization. In particular, race effects may be largely identified by differences across states with different racial and ethnic compositions. This confounding of race and place is an important issue receiving some recent attention in the literature [5-8]. We find that Georgia, Louisiana, and Detroit metropolitan Michigan have by far the greatest proportions of African Americans in these data (21%, 21%, and 17%, respectively) but no statistically significant difference between the propensity for blacks and whites to use mammography within these states. Other states with smaller proportions of blacks–namely CA and NJ (with 5% and 9%, respectively)–show significant differences between black and white utilization rates. However, looking across states with the 11-state average produced by the pooled model, results suggest that there are disparities in use between blacks and whites, which clearly contradicts what we find for the three states with the largest black populations. With this much heterogeneity across states, it is difficult to conclude anything about an average effect.

We also estimate a set of interaction effects between individual-level attributes and contextual factors in the woman's environment that have not been well studied. We are particularly interested in aspects of social support that might be captured by the interaction of a person's race/ethnicity with living in a more segregated place of the same race or ethnicity. The pooled model suggests a supportive effect for black women living in segregated black communities, but this is only found in a single state, NJ. The pooled model finds no supportive effect for Hispanic women living in segregated Hispanic communities, but CT, NJ, and NM all show significant interactions. The pooled model finds a negative effect on screening probability for Native Americans living in segregated Native American communities, but this is consistent with one state (NM) and inconsistent with two others (CA and IA).

Among the several contextual factors studied, commuter intensity, poor elderly English language ability, and elderly poverty have the greatest apparent impacts on mammography use; however, the impacts varied across the states studied. Pooling across states yields consistent results only when there is agreement across states in the sign of effect from the contextual covariate. In many cases, the pooled results can be misleading because states are quite heterogeneous. A recent review article uses meta-analysis to combine the results from various studies of disparities in mammography use conducted in specific regions of the United States with those that were nationwide in scope [55]. This meta-analytical approach imposes a great degree of statistical abstraction from the reality that places are quite different from one another, yielding average effect estimates across the pooled studies. This is similar to what happens when we pool states together, forcing the effect estimates to be the same everywhere. Pooling results in effect estimates that are an abstraction from reality, masking the fact that places are quite different. Comprehensive cancer control efforts should recognize that people and places exhibit a complex joint spatial distribution of characteristics. Efforts to reduce disparities must model the diversity in order to highlight the differences.

## Competing interests

The authors declare that they have no competing interests.

## Authors' contributions

LRM led the study and participated in all aspects of the study. T-MK led the multilevel analysis and contributed to the study design and writing. LC led the GIS data development and contributed to the writing. DD and LA contributed to data analysis and interpretation and the writing. LA also provided expert statistical and econometric consulting advice. All authors read and approved the final manuscript.
